# The Distinct Role of the Amygdala, Superior Colliculus and Pulvinar in Processing of Central and Peripheral Snakes

**DOI:** 10.1371/journal.pone.0129949

**Published:** 2015-06-15

**Authors:** Inês Almeida, Sandra C. Soares, Miguel Castelo-Branco

**Affiliations:** 1 Institute for Biomedical Imaging in Life Sciences (IBILI), Faculty of Medicine, University of Coimbra, Coimbra, Portugal; 2 Education Department, University of Aveiro, Aveiro, Portugal; University of Toyama, JAPAN

## Abstract

**Introduction:**

Visual processing of ecologically relevant stimuli involves a central bias for stimuli demanding detailed processing (e.g., faces), whereas peripheral object processing is based on coarse identification. Fast detection of animal shapes holding a significant phylogenetic value, such as snakes, may benefit from peripheral vision. The amygdala together with the pulvinar and the superior colliculus are implicated in an ongoing debate regarding their role in automatic and deliberate spatial processing of threat signals.

**Methods:**

Here we tested twenty healthy participants in an fMRI task, and investigated the role of spatial demands (the main effect of central vs. peripheral vision) in the processing of fear-relevant ecological features. We controlled for stimulus dependence using true or false snakes; snake shapes or snake faces and for task constraints (implicit or explicit). The main idea justifying this double task is that amygdala and superior colliculus are involved in both automatic and controlled processes. Moreover the explicit/implicit instruction in the task with respect to emotion is not necessarily equivalent to explicit vs. implicit in the sense of endogenous vs. exogenous attention, or controlled vs. automatic processes.

**Results:**

We found that stimulus-driven processing led to increased amygdala responses specifically to true snake shapes presented in the centre or in the peripheral left hemifield (right hemisphere). Importantly, the superior colliculus showed significantly biased and explicit central responses to snake-related stimuli. Moreover, the pulvinar, which also contains foveal representations, also showed strong central responses, extending the results of a recent single cell pulvinar study in monkeys. Similar hemispheric specialization was found across structures: increased amygdala responses occurred to true snake shapes presented to the right hemisphere, with this pattern being closely followed by the superior colliculus and the pulvinar.

**Conclusion:**

These results show that subcortical structures containing foveal representations such as the amygdala, pulvinar and superior colliculus play distinct roles in the central and peripheral processing of snake shapes. Our findings suggest multiple phylogenetic fingerprints in the responses of subcortical structures to fear-relevant stimuli.

## Introduction

It has been proposed that the amygdala responds preferentially to peripheral menacing stimuli [1]. However, relevant stimuli such as faces are preferentially processed in central vision [2] [3]. The amygdala seeks information from the eye region in human faces (e.g., [4] [5] [6] [7] [8]), receiving direct input from ventral areas (e.g., [9] [10] [11]) which are known to be biased to foveal (central) input [12]. Clinical evidence also shows that foveal vision deficits are reflected in face-selective regions [13]. The demonstration that distinct face-selective regions show stimulus-dependence and foveal bias is further corroborated by recent work combining intracranial EEG and eyetracking to study brain activity in temporal cortex and the functional specificity in the fusiform face (FFA) [14]. This work clearly shows that neural activity is mostly dependent upon the category of the foveated stimulus, with attention mechanisms filtering out the influence of objects surrounding the fovea in these face recognition regions [14].

Previously, we have extended this concept to the domain of affective processing of animal faces by showing that these activate the amygdala at a larger extent when presented at central, compared with peripheral spatial locations [15]. An important question is whether such foveal bias depends on the ecological relevance of objects and their components such as snake faces and their body shapes. It has often been hypothesized that resolution processing requirements influence organization of object representations [3] [12] and that stimuli not requiring fine-detailed analysis are efficiently processed by coarse peripherally-biased visual systems [3]. This later hypothesis is consistent with the idea that shapes do not require much detail to be processed [16] and can, therefore, be analysed in the visual periphery [12] [17]. Here, we follow the definition of Strasburger, Rentschler and Jüttner [18] who referred to central or foveal vision when an object falls within 2° of eccentricity, and to peripheral vision anything beyond 2° of eccentricity. A peripheral bias in medial regions, such as the anterior collateral sulcus in the parahippocampal gyrus, has been found for non-ecological stimuli that usually appear in the peripheral visual field (e.g., buildings) [3], and only require coarse processing of low spatial frequency (LSF) content. Studies of such biases need to take into account face/body dichotomies.

Classic studies in primates showed that learning of fear by observation of behaviour of conspecifics was facilitated when the fearful stimuli were reptiles, namely snakes, compared to other type of animals (e.g. rabbits) [19]. Snakes are prototypical stimuli characterized by their overall shape (and not specifically the face) and easily discriminated in the visual periphery as given by faster reaction times compared to other animal [20] and non-animal categories [21]. In line with these earlier findings it has been proposed that predation pressure from snakes is associated with the superior vision and larger brains in primates, which represented a critical facet of human evolution [22] [23] [24]. Thus, such stimuli are ideally suited to the design of our study and to test the face/shape-body dichotomy. Within the representation of living object categories, such as faces and bodies, there is indeed evidence that they are segregated in different brain regions. The face/body dichotomy is very well known in the literature, with face (FFA, STS, OFA) and body areas (EBA, FBA) being often explicitly mapped in brain imaging experiments (e.g. [25]), justifying the use of this stimulus dichotomy in experiments studying object representation central vs. peripheral biases. Nevertheless, snake face processing may be different as compared to other animals (including humans) face processing [e.g. 15].

Importantly, in addition to endogenous processes, individuals must capitalize on automatic, exogenous, attention mechanisms to potentially life-threatening events in order to allow safe avoidance or escape [26], regardless of whether they are central or peripheral. This could have shaped human vision to allow rapid identification of such stimuli [22]. A subcortical circuit comprising the amygdala [7] [27] [28], the superior colliculus (SC) and the pulvinar nucleus of the thalamus [29] [30] is thought to support rapid threat detection [7] [31] [32] [33].

However, although the fast visual detection of fear-relevant stimuli is an essential adaptive ability, the capacity to discriminate stimuli in the environment decreases with the physiological degradation of visual performance associated with retinal eccentricity [5] [34]. Behavioural studies with emotional stimuli do nevertheless suggest relatively good performance with parafoveal and peripheral locations both in recognition [12] [35] and categorization tasks for a wider category of stimuli, including faces. For instance, accurate animal detection is performed even at far peripheral locations [20] [36] and faces can be efficiently processed and detected when presented out of fixation (as efficient detection of peripheral faces/facial expressions seems crucial for survival) [37]. Nevertheless, brain imaging studies directly comparing central and peripheral vision point to a central processing bias for both human and animal faces [15] [38] [39]. Importantly, the ecological relevance of objects and their part components, such as the one given by distinct and particular features of faces and shapes has not been systematically addressed in neuroimaging studies. In fact, these have generally used emotional face stimuli presented in the central visual field (e.g. [40]). Besides these central versus peripheral issues, differences in the processing of ecological threat related stimuli between visual fields are reflected in hemispheric asymmetries and are known to occur in a general category of animals, with consequences for survival [41–44]. In humans, this has been tested using facial stimuli with reported right hemispheric dominance [45] [46].

The present study aims at investigating the role of spatial location in the amygdala and in other related subcortical structures such as the superior colliculus and pulvinar, when processing stimuli holding high evolutionary relevance, as it is the case with snakes [22] [47] and their specific shapes. Since the processing of snakes seems to be carried out independently of available resources [47–49], we tested whether their processing should occur outside the known central bias for (detailed) object recognition. Spatial location effects were studied both in terms of (as primary goal) central versus peripheral visual field locations and (as secondary goal) left vs. right visual hemifield asymmetries, due to the known right hemispheric preference for threat detection in a wide category of animals [50]. Additionally, it is important to probe how general such biases are, given that task instructions seem to change the neural correlates of threat processing [15] [51], with different neural correlates being attributed to explicit and implicit processing of fear-relevant information [29] [15] [51] [52]. Indeed, brainstem, and regions within the temporal lobe such as the superior temporal gyrus and the fusiform gyrus seem to be more implicated during explicit tasks [51], whereas the amygdala is in some contexts more involved in explicit [52] and others only during implicit processing [51]. Although this was not the major goal of our study it is important to test the generality of our findings using different task configurations. The explicitness of emotional processing can be varied by asking the participants to directly address the emotional cues (explicit task, e.g. name the emotional expression) or by focusing other aspects of the stimulus not directly related with emotion (implicit task, e.g. name specific nonemotional categories such as gender or age [51] [52]). Interestingly, attention to the ecologically relevant stimulus can be grabbed automatically (also called exogenous attention) by means of stimulus-driven mechanisms (e.g. ecological relevance, colour), or it can more voluntary be guided by goals of the observer using top-down mechanisms [37] [53]. Tasks in which target (emotional) and distractor (nonemotional) stimuli are not physically segregated employ endogenous mechanisms of attention [37] although they may remain implicit in terms of emotional focus. The amygdala is known to contribute not only in automatic and implicit processing [24] [29] [30] [51] but also to play a role in goal-directed and explicit processing [15] [52]. In parallel, the superior colliculus and pulvinar are involved both in automatic fear detection [29] [30] and in high level visual processing and attention [54–56], which motivated us to employ both tasks in the study. Interestingly, a recent tantalizing single-cell recording study in the pulvinar nuclei of monkeys by Van Le and colleagues [57] showed that its response is stronger and faster to (centrally-presented) snake stimuli, as compared to a broad range of stimulus categories.

To test these hypotheses we conducted an fMRI event-related design with manipulation of spatial location (central or peripheral) as the main outcome measure. Stimulus type (face, real or fake snake shape) was varied while using both implicit and explicit threat tasks.

## Materials and Methods

### Participants

Twenty participants with normal or corrected-to-normal vision (7 males, mean[SD] age 25.2[5.1]; education level range: 7–20) took part in the study. All individuals were right handed except one. Participants were selected from a pool of 94 individuals according to their scores on a Portuguese version of the Snake Phobia questionnaire (SNAQ) [58]. They were recruited both at the Faculty of Medicine, University of Coimbra, Portugal, and from a database of voluntary participants (http://voluntariosibili.pt.vu/). The mean[SD] rate of the fear scores in the snake phobia questionnaire was 13.65[8.83], ranging from 1 to 29 (maximum score = 30).

All participants gave written informed consent, according to the Declaration of Helsinki. The experimental protocol was approved by the ethics committee of the Faculty of Medicine of the University of Coimbra.

### Stimuli and apparatus

Pictures of snakes (faces and shapes) and stimuli resembling snake shapes (cables, strings, hoses, bracelets, trunks) were used as stimuli. The images were taken both from the internet and from the International Affective Picture System (IAPS) set (CSEA-NIMH, USA, csea.phhp.ufl.edu). The final set included 32 stimuli of each category, based on two pilot studies for stimuli selection. In the first pilot study (number of participants: n = 7), both true snakes shapes (number of pictures: n = 74) and snake faces (number of pictures: n = 74) were presented along with other animal faces (number of pictures: n = 24) and landscapes without animals (number of pictures: n = 24). In the second one (number of participants: n = 8), pictures of fake snake shapes (number of pictures: n = 96) were presented along with true snake shapes (number of pictures: n = 39) and natural landscapes without animals (number of pictures: n = 12). All stimuli were presented both in the right (RVF) and left visual fields (LVF). Both true snake shape and face pictures were selected based on faster reaction times (RTs) and accurate recognition, except for the fake snake shapes which were intended to have higher false alarm rates. In an additional experiment (number of participants: n = 20), the final set of 96 pictures was evaluated in terms of valence (range: -4 to 4) (snake faces: mean[SD] = -1.42[1.72]; snake shapes: mean[SD] = -1.49[1.35]; fake snakes: mean[SD] = 0.10[1.39]) and arousal (range: 0 to 8) (snake faces: mean[SD] = 4.46[2.01]; snake shapes: mean[SD] = 4.25[1.93]; fake snakes: mean[SD] = 1.48[1.95]) dimensions.

Each picture was presented within a squared shape, with the face or shape (true or fake) always centred, and yielding a visual angle of 6.84° x 6.84° (W x H). They were presented at one of three possible locations: centre, 0°, right or left, 7.71°. Importantly, the rational of choice for the visual angle was to select locations within (0°) and outside foveal vision (7.71°). In fact, the 5° eccentricity has been described as the limit of parafoveal vision [35] while others refer more generally to peripheral vision as any eccentricity higher than 2° of visual angle [18]. Although the main comparison addressed visual field locations, we also analysed stimulus spatial frequency spectra which were qualitatively similar.

Inside the scanner, the stimuli were back projected using an AVOTEC (http://www.avotec.org) projector on a 20(w) x 15(h) (1024 x 768 pixels) screen pad that was placed at a viewing distance of 50.5 cm by means of a head coil mounted mirror. The task was presented using Presentation software (Neurobehavioral Systems, USA, http://www.neurobs.com), and originally displayed on a monitor with a 60Hz refresh rate. Responses were given in a response box (Cedrus Lumina LP-400 response pad for fMRI, http://www.cedrus.com).

### Task design and procedure

**Fig 1:**
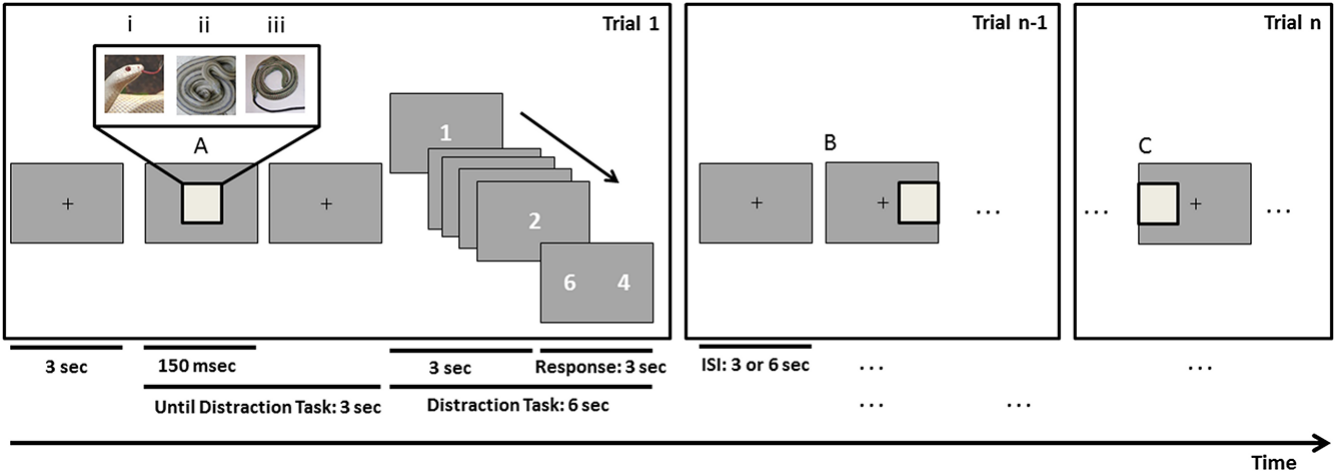
Experimental design. Each trial starts with a fixation cross followed by the presentation of a picture which can be of 3 different types, snake face (i), snake shape (ii), or fake snake shape (iii), and presented at 3 different locations in the visual display: centre (A), right (B) or left (C). Following the picture presentation (150 msec), time is given for a response according to the current task. After 3 seconds from stimulus onset, a distraction task starts to ensure maintenance of participants’ attention: counting the number of times number “1” appears and then selecting the right answer in a forced choice display. The inter-stimulus interval (ISI) is of 3 or 6 seconds until a new picture is displayed.

An fMRI event-related design was performed with 8 sequential runs of 36 trials each. Spatial location (centre, left, right) was manipulated as the main outcome measure. We additionally controlled for stimulus dependence (snake faces; true snake shapes; fake snake shapes). Importantly, the different tasks were performed while fixating a central cross. Participants were asked to report (a) if the picture presented was a real snake (either face or body) (task 1, implicit threat, first 4 runs) or (b) whether the stimulus represented threat (task 2, explicit threat, last 4 runs) by means of a 2-button (Yes/No) response box. Each trial started with a fixation cross (500 msec) followed by a picture presented at central, left (LVF) or right (RVF) position in the screen. Picture duration was kept short (150 msec) to prevent visual saccades and eye movements were recorded (MR compatible AVOTEC/SMI systems) to ensure central fixation. A distraction task, in which the participant was requested to count the number of “1”, appeared then in the screen, followed by a randomly presented inter-trial interval (ITI) (3 or 6 sec) (Fig 1). Participants were asked to remain as still as possible during the testing session. It was emphasized that this would be important to minimize data artefacts.

### Imaging data acquisition and preprocessing

Functional images were acquired in a 3T Siemens TimTrio scanner using BOLD contrast echo planar imaging (EPI, TR = 3 sec, TE = 30 msec, 40x3 mm-thick-slices, in-plane matrix 84 x 84 voxels, 165 volumes) covering the entire brain. The scanning session also included a high resolution T1 weighted anatomical scan (MPRAGE sequence, 1x1x1 mm3 voxel size, TR = 2.3 sec, TE = 2.98 msec, 160 slices) to help the transformation of the functional images into standard space. The data were preprocessed and analysed using BrainVoyager QX v2.6 (Brain Innovation, http://www.brainvoyager.com). Preprocessing included slice scan time corrections, temporal filtering and motion correction. Before group analysis, the images were spatially smoothed using a 6-mm full-width-half-maximum Gaussian kernel and then transformed into Talairach space.

### Statistical analyses

All the statistical analyses were performed using IBM SPSS Statistics 20 (IBM, USA, http://www.ibm.com/software/analytics/spss/) and Brain Voyager QX v2.6 software. The computation of effect sizes and power for the parametric analyses was performed with G*Power 3.1.6 [59]. For the non-parametric tests, effect sizes were computed based on the standardized statistics scores [60] using the following formula: $r=Z\sqrt{N}.$

### Behavioural data

Behavioural data were collected and trial responses were classified according to the following categories: correct, incorrect, misses or false alarms, for calculation of *d* prime measures (*d*’). Trials lacking response, trials corresponding to misses (e.g., snake faces or shapes not identified, snake faces or shapes not considered threatening) and false alarms (e.g., fake snake shapes identified as true snakes, or fake snake shapes considered as threatening) were excluded from the analyses but considered in the design model of the functional data analysis as confound predictors.

Observer’s *d* prime measures (*d*’) and reaction times (RTs) were obtained. The *d* prime, a measure of response sensitivity, was computed to test if there was a response bias [61] [62]. It was calculated for each task and spatial location by means of the following formula: *d*′ = *Z*(*hits*) − *Z*(*false alarms*). The idf.norm function of the IBM SPSS 20 software was used to compute the Z normalized scores for hits and false alarms. Due to the non-normal distribution of data, non-parametric tests were used for the *d* prime measure (Friedman [χ^2^F] and post-hoc Pairwise comparisons [t.s. stands for tests statistic], or Wilcoxon signed rank [T] tests). For the RT measure, we performed a Repeated Measures ANOVA 2x3x3 using task, spatial location and stimulus type as factors. When applicable, corrections of Greenhouse-Geisser were reported together with tests of sphericity. T-tests were used to identify statistically different pairs. All significant *p* values resulting from post-hoc tests were corrected for multiple comparisons.

### Functional data

Statistical analyses were performed using a RFX general linear model (GLM) approach. Event duration was set to 3 sec beginning in the stimulus onset. Both spatial location and stimulus type were manipulated, with 9 predictors (see below) being included in each single-subject’s design matrix. A box car function was defined for each predictor and convolved with a canonical hemodynamic response function.

Both regions of interest (ROIs) and whole brain analyses were then carried. First, two ROIs were defined in the left and right amygdalae of each participant using FreeSurfer v5.0.0 64-bit (http://surfer.nmr.mgh.harvard.edu/), automated segmentation of subcortical structures (http://surfer.nmr.mgh.harvard.edu/fswiki/SubcorticalSegmentation/). Anatomical brain FreeSurfer's processing includes removal of non-brain tissue, automated Talairach transformation, and segmentation of the subcortical white matter and deep grey matter volumetric structures. Amygdala ROIs in nifti (.nii) format where imported to BrainVoyager (plugin: nifticonverter_v106.dll) and transformed into Talairach space (mean[SD] coordinates, x, y, z, and number of voxels: left amygdala, -21.27[1.24], -3.23[1.33], -13.27[0.92], N = 3055 voxels; and right amygdala: 22.06[1.17], -3.90[1.50], -13.46[0.95], N = 3091voxels) (see S1 File.).

Parameter estimates (z-normalized beta weights, using specific volume segments to set the baseline) were computed for each ROI (subject-based) and each task, with ANOVAs RFX being performed using the IBM SPSS software. Planned RFX-GLM contrasts analyses were performed using BrainVoyager. Second, whole brain analyses were performed first for each task separately and then by directly comparing both tasks. The statistical maps display specific GLM contrasts using mask restriction (51377 voxels). Corrections for multiple comparisons were made with the Cluster Threshold plugin (BrainVoyager) using 1000 Monte Carlo simulations, with minimum cluster sizes corresponding to significance at a threshold of *p* < .05 for each contrast (initial voxel level threshold set to *p*<.05). Data were stored and are available in the Portuguese Brain Imaging Network repository (http://requestdata.ibili.uc.pt/).

## Results

### Behavioural data

#### Sensitivity index (d’)

In order to test if accuracy of performance was due to an increased/decreased bias to respond “yes” (see S1 Table), we have tested matched accuracy across tasks by using the bias free classical *d* prime measure. This measure computes the observer’s sensitivity to detect a signal while taking into consideration the false alarm rate (e.g., true snake, threat).

Differences were neither found to be significant between tasks (T = 132.000, Z = 1.008, *p* = .313) nor between tasks at each spatial location (task 1> task 2: centre, T = 96.000, Z = -.336, *p* = .737; left, T = 122.000, Z = .635, *p* = .526; right, T = 141.000, Z = 1.344, *p* = .179; 2-tailed tests). However, the sensitivity to detect a signal was found to depend on spatial location (task 1: χ^2^F(2) = 20.447, *p* = .000; task 2: χ^2^F(2) = 12.274, *p* = .002). Post-hoc tests revealed better performances for central presentations, compared with peripheral ones (task 1: centre > left, t.s. = .975, Z = 3.083, *p* = .006, r = .488; centre > right, t.s. = 1.350, Z = 4.269, *p* = .000, r = .675; right vs. left, t.s. = .375, Z = 1.186, *n*.*s*.; task 2: centre > left, t.s. = .800, Z = 2.530, *p* = .034, r = .400; centre > right, t.s. = 1.000, Z = 3.162, *p* = .005, r = .500; right vs. left, t.s. = .200, Z = .632, *n*.*s*.; 2-tailed tests).

#### Response time (RT) analysis

The analyses below include only correct responses (i.e., hits and correct rejections), which were considered for the RFX functional analyses. Separated analyses were performed for the two tasks (task 1: n = 20; task 2: n = 18) (see S2 Table).

A repeated measures ANOVA 2x3x3 revealed a main effect of spatial location (F_(2,34)_ = 9.222, *p* = .001, Cohen’s *d* = .737, power(1-β) = .817). Interaction effects were shown only between task and stimulus type (F_(1.306, 22.194)_ = 9.671, *p* = .003, Cohen’s *d* = .755, power(1-β) = .573, Mauchly’s W(2) = .468, *p* = .002, ε = .653).

Post-hoc paired samples *t*-tests showed the participants responded faster to centrally presented stimuli, as compared to peripheral ones (mean[SD] RT central = 866.95[150.67] msec; mean[SD] RT left = 916.52[169.70] msec; mean[SD] RT right = 944.71[162.19] msec; central < left: *t*_(17)_ = 3.204, *p* = .015, Cohen’s *d* = .755, power(1-β) = .923; central < right: *t*_(17)_ = 4.474, *p* = .000, Cohen’s *d* = 1.055, power(1-β) = .996).

For the interaction between task and stimulus type, a difference was found only for the fake snake (control) stimulus (task 1 > task 2: *t*_(19)_ = 4.426, *p* = .000 Cohen’s *d* = .982, power(1-β) = .995), with the participants being faster when judging its threatening content (task 2), as compared to deciding if the stimuli was a snake or not (task 1). Within task response time, differences between snake faces and fake snake stimuli were found only during task 1 (snake face < fake snake: *t*_(19)_ = 3.080, *p* = .018, Cohen’s *d* = .689, power(1-β) = .907).

### Functional data

#### Visual asymmetries in fear processing of ecological stimuli: Centre vs. periphery

Region of interest (ROI) analysis in the human amygdala: We were interested in testing the hypothesis that the amygdala processes snake faces preferentially in the centre and snake shapes (body parts) in the periphery, within each type task. We also aimed to assess if snakes fear scores, as given by the SNAQ questionnaire, predicted the activation levels in the amygdalae.

We first performed 3x3 ANOVAs RFX for each task (‘implicit threat’ snake identification or ‘explicit threat’ detection) in each amygdala ROI. Spatial location (centre, right visual field (RVF), and left visual field (LVF) and stimulus type (snake face, true snake shape, and fake snake control shape) were taken as within task factors. We then performed correlation analyses between the SNAQ scores and the significant contrasts given by the ANOVAs. All the post-hoc tests were corrected for multiple comparisons. During the ‘implicit threat’ task (task 1), a main effect of spatial location was found within both amygdalae (left: F_(2,38)_ = 6.594, *p* = .004, Cohen’s *d* = 2.258., power(1-β) = 1.000; right: F_(2,38)_ = 5.090, *p* = .011, Cohen’s *d* = 2.079, power(1-β) = 1.000). As for the ‘explicit threat’ detection task (task 2), a main effect of stimulus type was found for the left amygdala (F_(2,34)_ = 3.623, *p* = .038, Cohen’s *d* = 1.856, power(1-β) = 1.000). A marginal effect of spatial location was found for the left (F_(2,34)_ = 3.027, *p* = .062, Cohen’s *d* = 1.523, power(1-β) = 1.000) and for the right amygdala (F_(2,34)_ = 3.266, *p* = .050, Cohen’s *d* = 1.948, power(1-β) = 1.000).

**Fig 2:**
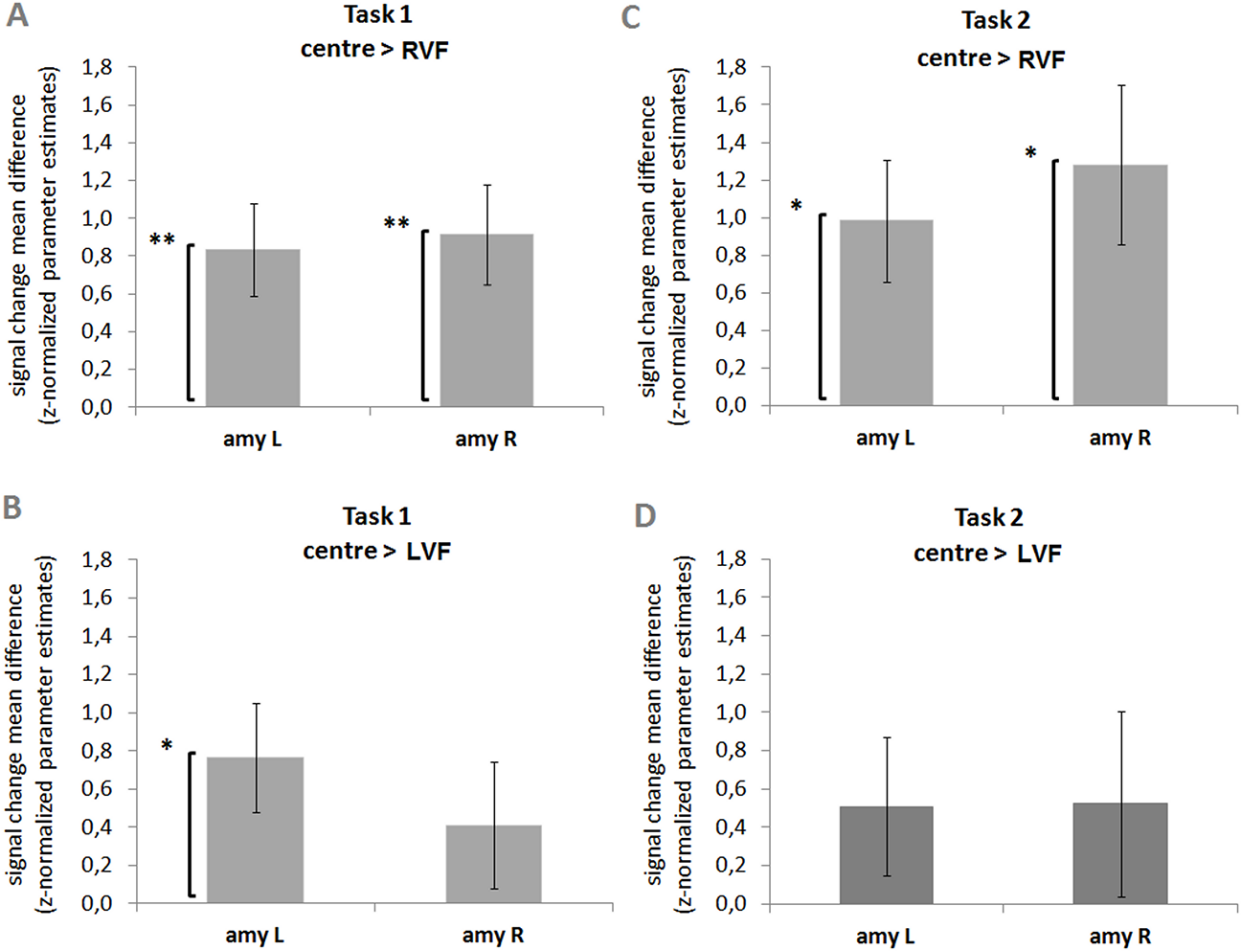
ROI amygdala: Effect of spatial location. Responses of the amygdala for centrally presented stimuli are larger than for RVF peripheral presentations, independent of task and amygdala. However, for the contrast ‘centre > LVF’, these differences depend both on task and amygdala: significant differences are found for the left amygdala during the implicit snake identification task (task 1), ROI RFX-GLM contrasts: mean differences in parameter estimates (z-normalized beta-values) for the contrasts ‘centre > RVF’, task 1 (**A**) and 2 (**C**), and for the contrasts ‘centre > LVF’, task 1 (**B**) and 2 (**D**), are displayed. Legend: LVF, left visual field; RVF, right visual field; amy L, amygdala left; amy R, amygdala right; *p**<.**05, ** p**<.**01. The bars display the standard error of the mean (SE).

**Fig 3:**
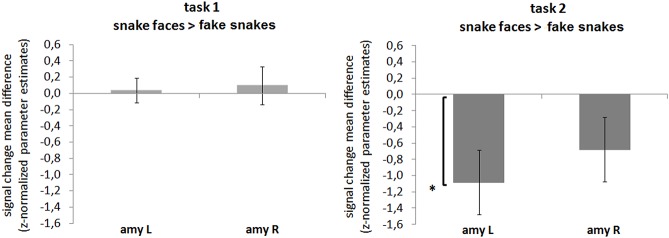
ROI amygdala: Effect of stimulus type. Responses of the amygdala to the snake faces are smaller than to the fake snake stimuli, showing differential decreased activation in a task and amygdala dependent manner. The left amygdala is preferentially involved in the discrimination of threatening and non-threatening stimuli during the threat detection task (task 2). ROI RFX-GLM contrasts: mean differences in parameter estimates (z-normalized beta-values) for the contrasts ‘snake faces > control fake snakes’, task 1 (**A**) and 2 (**B**) are displayed. Legend: amy L, amygdala left; amy R, amygdala right; *p**<.**05. The bars display the standard error of the mean (SE).

Post-hoc analyses following the ANOVAs main effects revealed that responses of the amygdala for centrally presented stimuli were larger than for RVF presentations, independent of task and amygdala ROI (task 1—left amygdala, centre > RVF: *t*_(19)_ = 3.396, *p* = .009, Cohen’s *d* = .759, power(1-β) = .948; right amygdala, centre > RVF: *t*_(19)_ = 3.472, *p* = .008, Cohen’s *d* = .776, power(1-β) = .955) (Fig 2). Moreover, for the contrast ‘centre > LVF’, these differences depended both on task and amygdala, with significant differences were found for the left amygdala during the implicit task (task 1—left amygdala, centre > LVF: *t*_(19)_ = 2.655, *p* = .047, Cohen’s *d* = .595, power(1-β) = .821). Figs 2 and 3 summarize the post hoc results.

Regarding the effect of stimulus type during task 2 (explicit) (Fig 3), left amygdala differences were found for the contrast ‘snake faces < fake snake shapes’ (*t*_(17)_ = 2.722, *p* = .045, Cohen’s *d* = -.641, power(1-β) = -.833), which seems better explained by diminished responses to faces particularly during the explicit task, as compared to fake snake shapes.

The range of scores (1–29) of our participants in the snake fear questionnaire (SNAQ), enabled us to test if it was related to the activity patterns observed in the amygdala. For the implicit task, the contrast ‘central > RVF’ in the right amygdala revealed a significant positive correlation with the SNAQ scores (r = .55, *p* = .012).

#### Whole brain RFX analysis to inspect the role of SC and pulvinar as a function of spatial location

**Fig 4:**
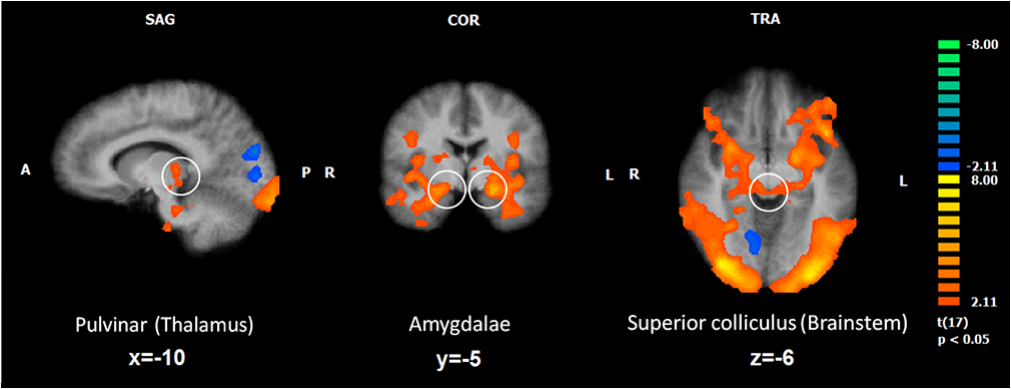
Whole brain: ‘Central vs. Peripheral’ spatial location. Activated regions yielded by the RFX group analysis for the contrast ‘centre > periphery’. Bilateral amygdala (central panel), pulvinar nucleus of the thalamus (left) and superior colliculus in the midbrain (right) show larger responses to central compared with peripheral presentations. Voxel level threshold was set at p< .05, followed by a cluster threshold correction set at p<.05 which resulted in a minimum cluster size of 170 voxels.

Whole brain RFX contrasts were also performed to identify brain regions showing spatial location effects. The results are summarized in Fig 4 (brain regions, peak voxel coordinates and statistics are presented in S3 and S4 Tables).

The contrasts computed for the effect of spatial location (pooled tasks) revealed and confirmed mainly regions with increased activity for central presentations. As expected, regions in the occipito-temporal ventral stream activated more for central presentations (e.g. bilateral inferior and left middle occipital gyrus, left occipital and temporal-and right temporal fusiform gyrus). Importantly, we found increased central bilateral amygdala activation and right uncus, together with the insula (bilateral), the nucleus of the thalamus (e.g. pulvinar), and the basal ganglia (e.g. right putamen, right caudate body). Additionally, we found increased activation in the brainstem (e.g. midbrain including SC) and the cerebellum.

We found only two regions in the occipital cortex which, as expected, activated more for the periphery than for central stimulation: the cuneus (bilaterally) and the (right) lingual gyrus. A part of the cerebellum (culmen) also showed increased peripheral activity (see Fig 4 and S3 Table).

In order to explore different neural correlates depending on the task instructions, we compared performance within the implicit (n = 20) and explicit tasks (n = 18) between central and peripheral spatial locations.

Implicit task (task 1): stronger activity was found in the amygdala bilaterally, basal ganglia (caudate head and putamen, lateral and medial globus pallidus) and hippocampus besides regions within the frontal, temporal and parietal lobes during central processing of snake stimuli. Occipital, frontal and parietal regions also showed clusters of stronger activity for peripheral stimuli (S4 Table).

Explicit task (task 2): bilateral amygdala, and basal ganglia (putamen, lateral globus pallidus) were found to be preferentially engaged during central processing. Importantly, insula, brainstem and thalamus nuclei (pulvinar and medial dorsal nucleus) activated more for central stimuli but only during the explicit task. In contrast with the implicit task, no regions showed preferential activation during peripheral representations (S4 Table).

#### Visual asymmetries in fear processing of ecological stimuli: Left vs. right evolutionary asymmetries. Region of interest (ROI) analysis in the human amygdala to test hemispheric asymmetries as a function of Stimulus type

**Fig 5:**
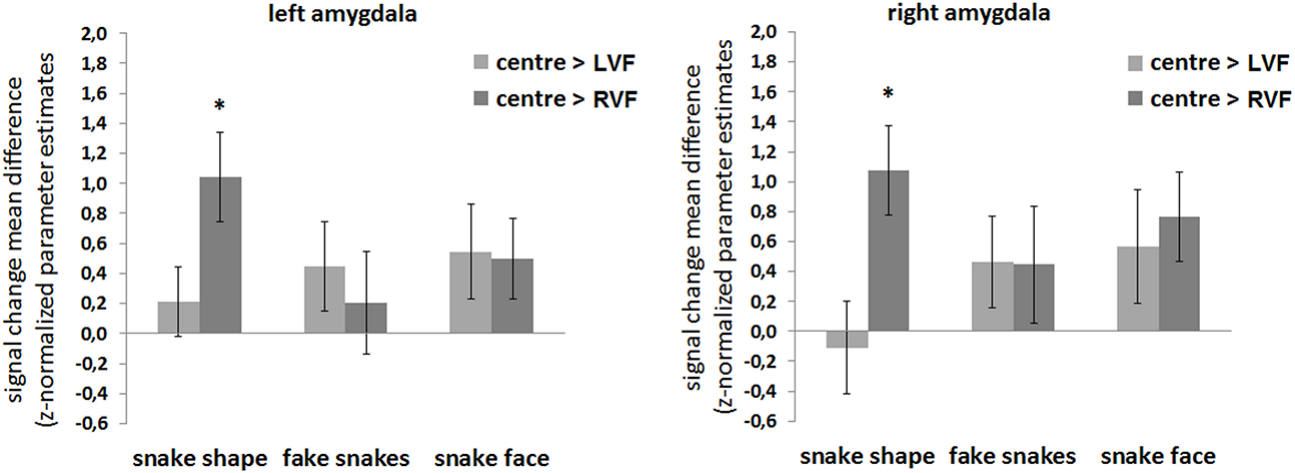
ROI amygdala: Effect of visual field and stimulus type. Hemispheric asymmetries are found in a stimulus type dependent manner, as response difference between the centre and each of the visual hemifields (LVF, RVF) are specific for the true snake shapes. ROI RFX-GLM contrasts: mean differences in parameter estimates (z-normalized beta-values) for the contrasts ‘centre > LVF’ and ‘centre > RVF’, for the right and the left amygdala ROI. Legend: LVF, left visual field; RVF, right visual field; *p**<.**01, corrected for multiple comparisons. The bars display the standard error of the mean (SE).

**Fig 6:**
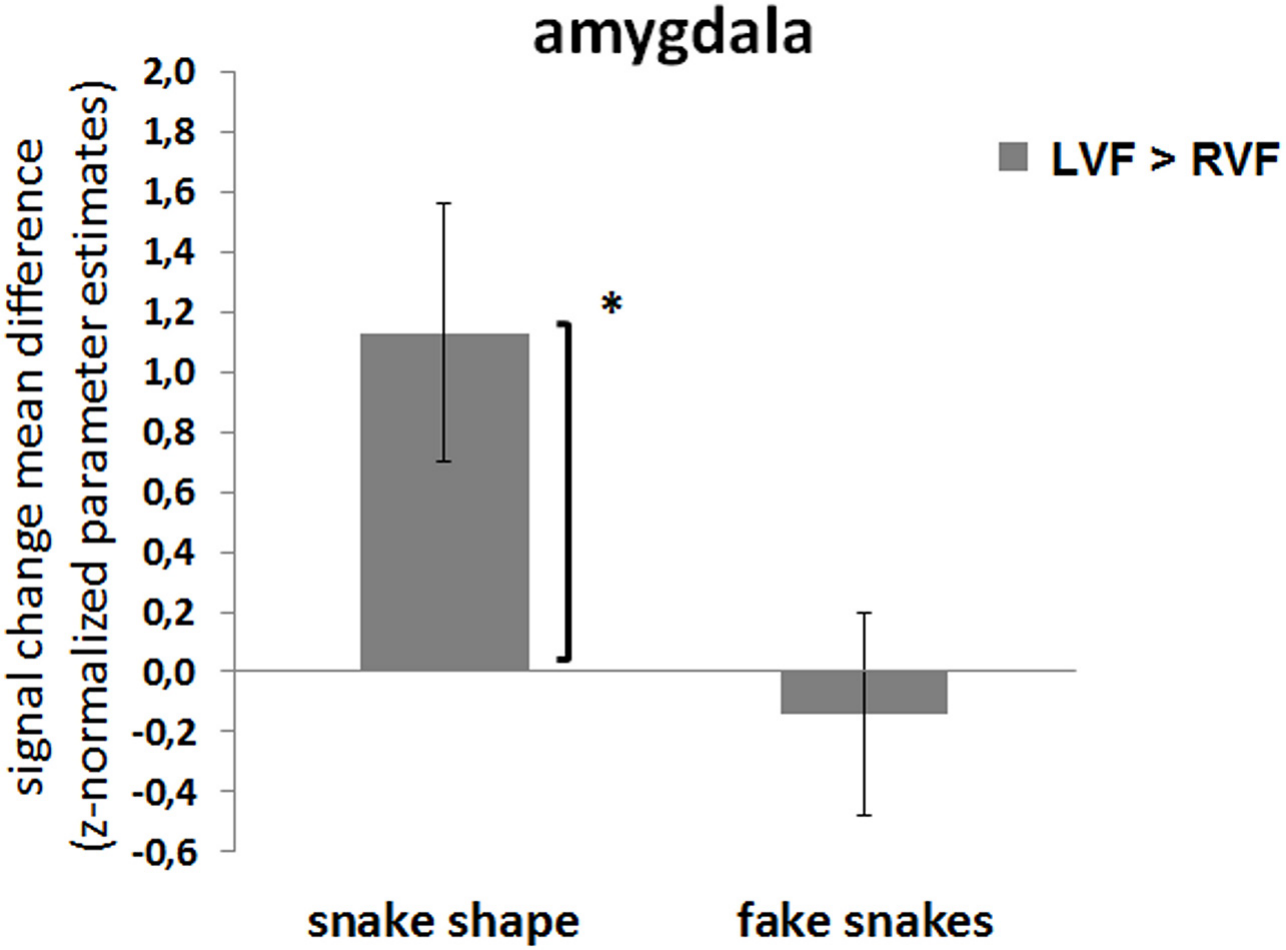
ROI amygdala: Effect of visual field and the specificity of the true snake shape stimuli. Hemispheric asymmetries are found in a stimulus type dependent manner—the right hemisphere (left visual field) responds more to true snake shapes, but the same in not found for the fake snakes (control stimuli). ROI RFX-GLM contrasts: mean differences in parameter estimates (z-normalized beta-values) for the contrasts ‘LVF > RVF’, for the amygdala (left and right ROIs together). Legend: LVF, left visual field; RVF, right visual field; *p<.05, corrected for multiple comparisons. The bars display the standard error of the mean (SE).

The above pattern of results suggested the existence of hemispheric asymmetries regarding the spatial location of the stimulus. Given the ecological value of snake stimuli, we hypothesized the presence of hemispheric asymmetries as a function of stimulus type. The sections below explore this hypothesis, with results being summarized in Figs 5 and 6. They basically show that snake shapes (perceived as true) are processed differently as compared to faces and fake snakes, with a clear hemispheric bias.

We found a significant effect of lateralization that was specific for the true snake shapes stimuli and was absent for snake faces or fake snakes (pooled tasks). Accordingly, for the snake shapes, differences between the centre and the RVF (left hemisphere, LH) occurred for both the left (*t*_(17)_ = 3.522; *p* = .003, Cohen’s *d* = .829, power(1-β) = .958) and the right (*t*_(17)_ = 3.595; *p* = .002, Cohen’s *d* = .848, power(1-β) = .964) amygdalae, whereas no significant effects were found to the contrast centre > LVF (which maps to the visually dominant right hemisphere, RH). Importantly, significant effects were found neither for the snake face nor for the fake snake stimuli (Fig 5).

These results suggest the occurrence of hemispheric asymmetries specific for the snake shape stimuli judged as true snakes. As this particular effect seemed to be independent of the amygdala side, we concatenated the two ROIs and tested the effect of hemispheric asymmetries by comparing the amygdala response for LVF and RVF presentations. In fact, the amygdalae responded significantly more to (attentionally / emotionally dominant) LFV than to the RVF presentations of true snake shapes (LVF > RVF: *t*_(17)_ = 2.617, *p* = .018, Cohen’s *d* = .617, power(1-β) = .807). The same was not true for the fake snake controls (LVF > RVF: *t*_(17)_ = -0.416, *n*.*s*.; 2-tailed, corrected for multiple comparisons) (Fig 6).

#### Whole brain RFX analysis to test hemispheric asymmetries in SC and pulvinar

**Fig 7:**
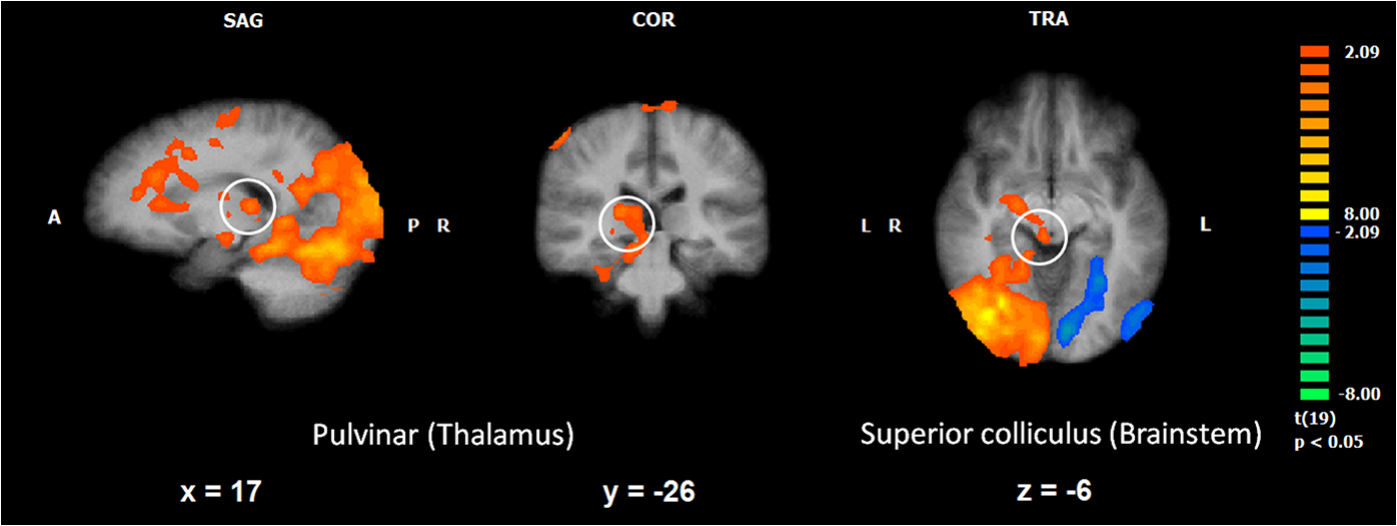
Whole brain: Effect of visual field ‘LVF > RVF’. Activated regions yielded by the RFX group analysis for the contrast ‘LVF > RVF’. A hemispheric asymmetry was found particularly in the right pulvinar and in the right superior colliculus for LVF presentations of snake stimuli, during the implicit snake identification task, task 1 (n = 20). Legend: LVF, left visual field; RVF, right visual field. Voxel level threshold was set at p<.05, followed by a cluster threshold correction set at p<.05 which resulted in a minimum cluster size of 166 voxels.

We investigated if other two main structures involved in the subcortical pathway, the pulvinar and the SC, also showed a LVF bias. For the implicit task alone, significant (for the contrast LFV > RVF, independent of stimulus type) clusters (corrected, minimum cluster size = 166 voxels) were found both in the right pulvinar (number of voxels = 1156; peak voxel: *t*_(19)_ = 4.079, *p* = 0.00064; x = 17, y = -26, z = 12) and in the right SC (number of voxels = 1461; peak voxel: *t*_(19)_ = 3.635, *p* = 0.00176; x = 8, y = -26, z = 9) (Fig 7).

## Discussion

The present study aimed to investigate the role of spatial location in subcortical structures, in particular the amygdala, in the processing of stimuli holding high evolutionary relevance, such as snakes. In particular, we asked whether visual processing in these regions depends on central vs. peripheral location or LFV vs. RVF. We were also interested on the dependence of ecological nature of the stimuli (true or false snake body shapes or snake faces), and whether these effects could be replicated in different tasks types (implicit and explicit). A pivotal question, given the primary question of the role of spatial location, was whether the same central bias as previously observed for fear-relevant faces [15] would be found for snake stimuli or instead a peripheral bias was to be expected. Moreover, by performing whole brain analyses, we aimed to identify which regions were dependent on spatial location manipulations as a function of the features of these evolutionary fear-relevant stimuli.

### Visual asymmetries in fear processing of ecological stimuli

#### Centre vs. periphery: the relevance of snake ‘body shape’ vs. ‘faces’ features

Amygdala ROI analyses showed a main effect of spatial location with larger responses for central presentations bilaterally. Of particular importance, (true) snake shapes showed a specific central bias, for both amygdalae, unlike the other stimulus types (snake faces and fake snakes). These findings confirm that these ecological stimuli are processed in a special manner. The central bias is consistent with the notion that amygdala receives strong input from foveally dominated ventral stream occipito-temporal areas [9] [10] [11] with strong responses to human faces [3]. The data presented here extends previous findings that the amygdala is recruited by animal face processing during explicit evaluations [15] [52].We had expected that snake shapes (body parts) would engage more the amygdala during peripheral presentations (LVF or RVF) which more often reflects implicit processing than central vision, because foveal vision more frequently reaches visual awareness, although not in a strict manner [63–65]. In addition, and as with other stimuli based in LSF cues, snake shapes were also predicted to be preferentially processed in areas devoted to peripheral processing (e.g., collateral sulcus) [3]. Surprisingly, we found that snake-related stimuli activate more strongly central representations, thus showing that amygdala responses to snake-related stimuli benefit as much of central vision as other stimuli which show foveal bias, such as faces [15]. In contrast to the “building-type” stimuli reported by Levy and colleagues [3], true snakes are fear-relevant stimuli [47] [66] [67] with high ecological value [22] and may be prioritized for central processing, which is in any case a feature of primate vision [22].

Given the surprising response patterns observed for the snake faces, our interpretation is that snake shapes are ecologically more relevant than snake faces (which may even lead to decreased levels of activation in the explicit threat task) and benefit from prioritized and detailed central processing in foveal regions. Contrary to human and other animal faces, snake faces are stimuli which humans are not used to foveate. This might explain why our main findings imply specialized processing of true snake shapes rather than faces, as this stimulus display less information than other animal faces [15]. Accordingly, body shape features convey the necessary and sufficient information concerning potential fear-relevant content [22] rather than the face.

We believe that our results may help reconcile different views regarding the role of the amygdala in emotional processing. Although this structure is sometimes proposed to have a preferential role in non-conscious, implicit, emotional processing through a subcortical pathway [30] [67], others postulate its dual role in both preattentive and conscious appraisal processes through rich subcortical and cortical connections [55]. We actually believe that both possibilities are rendered viable by our results: preattentive detection of fear-relevant stimuli is preferentially made through a coarse (LSF tuned) pathway [67], with our results suggesting that the amygdala may also be recruited for conscious and central processing of ecological fear-relevant stimuli. Accordingly, we showed that central presentations of snake-related stimuli actually elicited stronger responses in the amygdalae, particularly when compared to RVF presentations, independent of implicit or explicit nature of the task (although the effects of spatial location are more evident in the implicit task). Interestingly, for the contrast centre > LVF (which corresponds to the emotionally / attentionally dominant RH) [68–71] these differences were less evident, in line with the lateralization hypothesis (but see [72]). LVF/RH dominance was confirmed by direct comparison of hemifield responses (see following section). In other words, both central and dominant RH peripheral representations are relevant during ecologically-relevant stimuli processing.

Interestingly, the centre > RVF difference in the right amygdala for the implicit task was correlated with reported fear in our participants (as measured by the Snake Fear questionnaire, SNAQ), but not the centre > LVF difference. This is consistent with the notion that both central and LVF (right hemisphere) processing are important for this particular stimulus type but not RVF (left hemisphere). Moreover, this suggests that the more fearful (of snakes) a participant is, the larger the central modulation. We discuss these results in two ways. First, asymmetries centre > RVF and centre > LVF are distinctly patterned in the amygdala, with RVF presentations not being as efficient as LVF presentations to elicit a response in the amygdala, particularly for (true) snake shape stimuli. These differences are accommodated by LVF > RVF asymmetries suggesting a right hemispheric dominance in fear-relevant stimulus detection. Therefore, the correlation of the difference centre vs. RVF (left hemispheric dominance) with the SNAQ score suggests a less influent role of the left hemisphere in fear related behaviour [73]. Second, central presentations elicit stronger amygdala responses than presentations to the RVF (left hemisphere), and this is positively correlated with fear for snakes. Previous research has suggested that the amygdala is especially involved under non-conscious appraisal of fear-relevant stimuli, and inhibition of the fear module may be supressed by prefrontal networks during conscious appraisal [67] [74], which our results seem to extend. In fact, it seems natural that objects that we fear (e.g. snakes, spiders) elicit stronger anxiety when in our focus of gaze (central processing). Accordingly, direct gaze towards the feared stimulus elicits stronger anxiety, even if the task is not explicit, and is in the base of phobic avoidance of the feared stimulus [67] [75–79]. Additionally, since this occurred particularly during the snake identification task, the participants had to more effortfully focus on detailed snake-related information. In sum, we speculate that implicit processing (and particularly, discriminating real from fake snakes) does probably reflect stronger trait-related aspects regarding fear of snakes in amygdala responses than explicit processing. The latter (explicit task) implies cognitive control and less likely to reflect individual responses.

Interestingly, whole brain analyses provided evidence of both central dominance and explicit processing bias in other threat-related subcortical structures. More specifically, we found an interesting pattern of activation within the brainstem, particularly in the SC, with strong responses for explicit processing at central locations, and for the pulvinar nucleus of the thalamus, which also responded more to central presentations than to peripheral ones.

The SC is classically implicated in attentional guidance to relevant positions in our environment [80]. Moreover, the role of SC neurons representing the fovea and active during fixation behaviour [81] may be important to keep attention focused, which may help explain why we found increased activity for centrally presented stimuli. This suggests that this structure is also related to the foveal processing of affective stimuli. Remarkably, we found strong responses of the SC in particular for explicit tasks at central fixation. This extends the current knowledge of the role of the SC in emotionally-related processes and emphasizes its role during both explicit and central processing of stimuli requiring foveation and fixation. Our findings are also consistent with the known magnification factors of foveal input in the SC, particularly in its rostral part [82].

As for the pulvinar, we found stronger activity for central presentations of snake related stimuli, irrespective of task. The role of pulvinar in attention related processes is well known, constituting a higher centre directly connected with the SC for attentional guidance and high level visual processing [54–56]. A recent paper by Saalman and colleagues [83] demonstrated that the pulvinar synchronizes activity between interconnected cortical areas according to attentional allocation. This suggests a critical role for this thalamic structure not only in attentional selection but more generally in regulating information transmission of high level features across the visual cortex [84]. This adds to the view that the pulvinar may also be involved in regulating information transfer during more automatic, preattentive processes [29] [85]. Such multiple facets of pulvinar function are consistent with the proposal that different pulvinar parts might have distinct functional roles [86]. Recently, Van Le and colleagues [57], following Isbell’s theory on the evolutionary relevance of snake stimuli [22] [23], performed single-cell recording in the pulvinar nuclei of monkeys and showed that its response was not just stronger but also faster to centrally presented snake stimuli, as compared to angry and neutral faces, hands, and simple geometric shapes. This study further supports the potential evolutionary weight that snakes might have had in the development of the primate visual system.

Our findings clearly add to the notion that both the SC and pulvinar process implicit emotional content [30]. Moreover, they are also consistent with findings of increased brainstem response (including the SC) during explicit, and central, appraisal of faces [52] and increased pulvinar activity in central processing of facial expressions [51] or accurate conscious perception of a stimulus (hit vs. miss contrast) [40].

Interestingly, we found activity within the middle temporal gyrus during the performance of both tasks, consistent with previous studies showing its involvement during tasks of covert attention (central fixation while monitoring the periphery) [87] [88] [89]. Nevertheless, we found that during the implicit task, the MFG activated more during the peripheral processing, what was expectable, whereas during the explicit task, it activated more for central presentations. A potential interaction with emotional content and focus of task might be hypothesized and deserves additional studies.

#### Left vs. right evolutionary asymmetries in true snake body perception

The amygdala (both left and right) showed a bias towards true snake shapes presented to the LVF (RH), compared with same stimuli presentations to the RVF (LH). This effect was neither observed for fake snakes nor for snake face stimuli. This is consistent with the amygdala dominance both for fear-relevant stimuli [66], as well as with its bihemispheric left-hemifield (LVF) superiority in visuospatial attention [45] and emotionally-relevant domains [46].

We predicted that fear-relevant shapes should be processed in a bottom-up, stimulus-driven manner. This mechanism expected for fear-relevant stimuli is related to visual orienting, also termed exogenous attention [90] [91] and is often associated with right hemispheric dominance [68] [90], which we did observe. Nevertheless an endogenous component cannot be excluded. These findings are consistent with previous research in animal behaviour, showing the existence of evolutionary hemispheric asymmetries for threat detection and the launching of appropriate responses [41] [42] [50]. Evidence shows that attention to global cues and predator detection is (right) lateralized for instance in toads, fishes, birds [41] [42], dogs [44], and humans, for whom a RH bias for processing emotional items has been reported [92], particularly of negative pictures [46] [93] [94].

Importantly, contralateral activity both in the pulvinar and in the SC was found only for LVF (RH dominance) presentations of snake stimuli (irrespective of type) during the implicit threat task. Previous reports showed a RH (LVF) bias within the amygdala, superior colliculus and pulvinar, particularly for the contrast fearful vs. neutral faces [46]. These results support evolutionary views and reported hemispheric asymmetries in spatial attention concerning the pulvinar [95].

### Limitations

We found clear evidence that the same structures may support explicit and implicit processing mechanisms. However it is important to note that the particular weight of central vs. peripheral responses may depend on the timescale and task requirements. Although we found increased central responses in the amygdala, pulvinar and SC to snake stimuli, it is possible that transiently enhanced early onset responses for peripheral stimuli occur at a time scale not detectable by the temporal resolution of our method [38]. In any case, strong responses were also found in the left (dominant) peripheral hemifield.

An important limitation intrinsic to the experimental design of this study is that the main outcome variable (spatial location) was studied as a within task variable. This is because once an explicit instruction is given, true implicit processing is no longer possible in repeated presentations. This is known as interference or carry over effect that cannot be solved by counterbalancing because it "carries over" from one experimental condition to another (for a critical review see [96]). Therefore, direct comparisons between tasks were not feasible and this is why we prioritized within task effects. In fact, our main goal was to compare central and peripheral processing of threat information while using both implicit and explicit tasks to control for the nature of the task. Importantly, it is relevant to consider that “implicit emotional” tasks do not necessarily mean “implicit attention”.

Another limitation of this study is that we used only static visual stimuli, instead of including moving snakes which are known to elicit both greater physiological and self-reported arousal compared to static ones [97]. Nevertheless, Courtney and colleagues [97] have cautioned for the case of selective sensitization to the stimuli. They pointed that major differences might arise from the stimuli being contrasted, since the videos’ high effectiveness in inducing fear might render other types less effective. In our study, as in other emotional studies (e.g. [98]) the same type of stimuli was used in both the conditions being contrasted. Therefore contrast analyses are not affected by choice of dynamic vs. static stimuli.

## Conclusions

We found that the superior colliculus, pulvinar and amygdala respond to evolutionary relevant stimuli with a surprising central bias. This pattern was more salient for true body snake stimuli, as for other animal faces but not snake faces. These findings also extend a previous single cell study in monkeys [57] which suggested an important role of foveal vision. Importantly, the phylogenetic pattern of peripheral of RH dominance for fear-relevant stimuli, and specifically true snake shapes, was preserved. Moreover, we found evidence for important SC responses to foveal fear-relevant stimuli in line with the notion that a large foveal representation and a population of fixation-related neurons are present in this structure in primates. A similar effect was found in the pulvinar suggesting an important role for the control of information transfer, which occurs for explicit and not only for implicit processing.

In our view, these results suggest that mechanisms of central and peripheral vision share the same neural structures irrespective of task. These may serve different purposes, as a function of the ecological relevance of the stimuli (true snake body shapes vs. false shapes or snake faces). This interpretation helps reconcile opposing views in terms of the functions of the amygdala pulvinar and superior colliculus, emphasizing the core role of these structures in routing fear-relevant information, independently of whether it is implicitly or explicitly routed according to attentional demands.

## Supporting Information

S1 File Regions of Interest (ROIs) in the right and left amygdala. Resulting Regions of Interest (ROIs) defined in the right and left amygdala of each one of the participants following FreeSurfer v5.0.0 64-bit (http://surfer.nmr.mgh.harvard.edu/) automated segmentation of subcortical structures. Mean coordinates (mean x [SD], mean y [SD], mean z [SD]) of all Regions Of Interest defined for the 20 participants were 22.06[1.17], -3.90[1.50], -13.46[0.95] for the right amygdala (number of voxels = 3091), and -21.27[1.24], -3.23[1.33], -13.27[0.92] for the left amygdala (number of voxels = 3055).
(PDF)

S1 Table Sensitivity index (d’) (mean[SD]) as a function of Spatial location and Stimulus type. (PDF)

S2 Table Response time (mean[SD]) as a function of Spatial location and Stimulus type. (PDF)

S3 Table Summary of random-effects (RFX)-GLM for the factor Spatial location. (PDF)

S4 Table Summary of random-effects (RFX)-GLM for Spatial location within Task. (PDF)
