# Correction: The Distinct Role of the Amygdala, Superior Colliculus and Pulvinar in Processing of Central and Peripheral Snakes

**DOI:** 10.1371/journal.pone.0141175

**Published:** 2015-10-15

**Authors:** Inês Almeida, Sandra C. Soares, Miguel Castelo-Branco

The following information is missing from the Funding section: This study was supported by the Portuguese Foundation for Science and Technology (FCT) PEST UID/NEU/04539/2013 (CNC.IBILI).
