# Supplementary material for: The Distinct Role of the Amygdala, Superior Colliculus and Pulvinar in Processing of Central and Peripheral Snakes
Source: PLoS One. 2015 Jun 15;10(6):e0129949. doi: 10.1371/journal.pone.0129949 (PMC4467980; doi:10.1371/journal.pone.0129949)
Supplement: S1 File — Resulting Regions of Interest (ROIs) defined in the right and left amygdala of each one of the participants following FreeSurfer v5.0.0 64-bit (http://surfer.nmr.mgh.harvard.edu/) automated segmentation of subcortical structures. Mean coordinates (mean x [SD], mean y [SD], mean z [SD]) of all Regions Of Interest defined for the 20 participants were 22.06[1.17], -3.90[1.50], -13.46[0.95] for the right amygdala (number of voxels = 3091), and -21.27[1.24], -3.23[1.33], -13.27[0.92] for the left amygdala (number of voxels = 3055). (PDF) [file pone.0129949.s001.pdf]

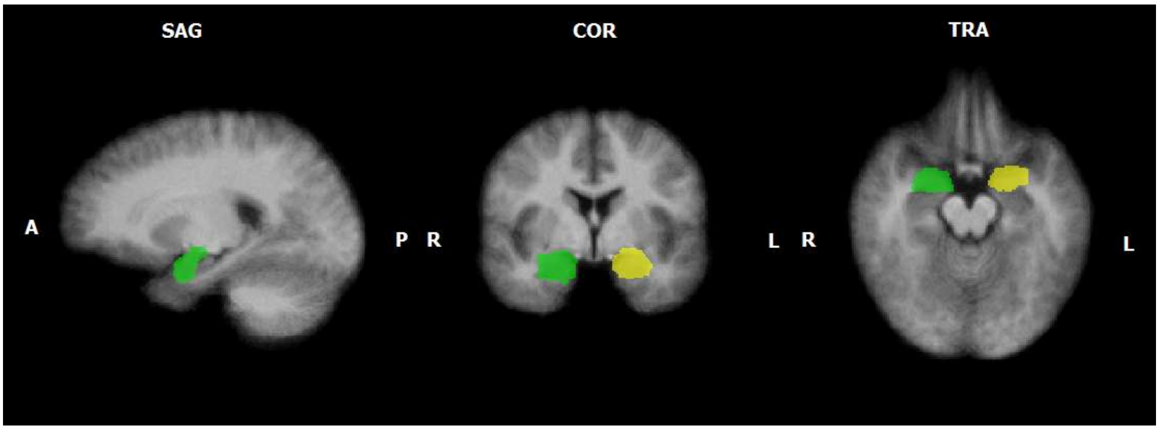

S1 Fig. – Regions of Interest (ROIs) in the right and left amygdala. Resulting Regions of Interest (ROIs) defined in the right and left amygdala of each one of the participants following FreeSurfer v5.0.0 64-bit (http://surfer.nmr.mgh.harvard.edu/) automated segmentation of subcortical structures. Mean coordinates (mean x [SD], mean y [SD], mean z [SD]) of all Regions of Interest defined for the 20 participants were 22.06[1.17], -3.90[1.50], -13.46[0.95] for the right amygdala (number of voxels = 3091), and -21.27[1.24], -3.23[1.33], -13.27[0.92] for the left amygdala (number of voxels = 3055).
