# Supplementary material for: The Distinct Role of the Amygdala, Superior Colliculus and Pulvinar in Processing of Central and Peripheral Snakes
Source: PLoS One. 2015 Jun 15;10(6):e0129949. doi: 10.1371/journal.pone.0129949 (PMC4467980; doi:10.1371/journal.pone.0129949)
Supplement: S1 Table — (PDF) [file pone.0129949.s002.pdf]

S1 Table. Sensitivity index (d') (mean[SD]) as a function of Spatial location and Stimulus type: for the "implicit threat" snake identification task (Task 1) and for the "explicit threat" detection task (Task 2).

Task	Left mean[SD]	Centre mean[SD]	Right mean[SD]	total
**Snake identification (implicit task)**	***2,64***[2,44]	***5,01***[3,47]	***1,72***[1,69]	**2,09**[1,65]
**Threat detection (explicit task)**	***3,84***[2,99]	***4,90***[3,14]	***3,17***[3,05]	**2,80**[2,44]
**total**	**3,24**[1,74]	**4,96**[2,44]	**2,44**[2,02]	
