# Supplementary material for: The Distinct Role of the Amygdala, Superior Colliculus and Pulvinar in Processing of Central and Peripheral Snakes
Source: PLoS One. 2015 Jun 15;10(6):e0129949. doi: 10.1371/journal.pone.0129949 (PMC4467980; doi:10.1371/journal.pone.0129949)
Supplement: S2 Table — (PDF) [file pone.0129949.s003.pdf]

S2 Table. Response time (mean[SD]) as a function of Spatial location and Stimulus type (hits and correct rejections only)**:** for the "implicit threat" snake identification task (Task 1) and for the "explicit threat" detection task (Task 2).

Task	Left (LVF) mean[SD]	Centre mean[SD]	Right (RVF) mean[SD]	
Snake identification (implicit task)				**total**
Snake faces	***916,26***	***820,31***	***955,67***	**897,42**
	[237,93]	[200,75]	[224,48]	[207,60]
Snake shapes	***928,32***	***858,08***	***983,10***	**923,17**
	[248,26]	[212,81]	[225,01]	[216,91]
Fake snakes	***1004,64***	***969,35***	***1014,00***	**996,00**
	[178,56]	[176,57]	[205,15]	[173,71]
**total**	**949,74**	**882,58**	**984,26**	
	[200,56]	[180,57]	[193,85]	
Threat detection (explicit task)				**total**
Snake faces	***971,99***	***901,23***	***929,27***	**909,48**
	[349,06]	[275,48]	[207,36]	[235,30]
Snake shapes	***992,53***	***866,08***	***977,37***	**929,53**
	[306,85]	[159,19]	[221,43]	[187,61]
Fake snakes	***865,87***	***868,56***	***879,72***	**888,36**
	[142,47]	[138,89]	[151,06]	[184,83]
**total**	**945,27**	**872,46**	**931,07**	
	[230,26]	[171,54]	[169,74]	
