# Supplementary material for: The Distinct Role of the Amygdala, Superior Colliculus and Pulvinar in Processing of Central and Peripheral Snakes
Source: PLoS One. 2015 Jun 15;10(6):e0129949. doi: 10.1371/journal.pone.0129949 (PMC4467980; doi:10.1371/journal.pone.0129949)
Supplement: S3 Table — (PDF) [file pone.0129949.s004.pdf]

S3 Table. Summary of random-effects (RFX)-GLM for the factor Spatial location: contrasts, outputs and statistics.

Region	Peak X (TAL)	Peak Y (TAL)	Peak Z (TAL)	Nr of voxels	*t*	*p*
**Centre > Periphery**						
R occipital (cuneus)	20	-95	0	1678	6.894	0.000003
L occipital (cuneus)	-27	-92	-2	345	7.248	0.000000
R occipital fusiform gyrus	29	-84	-12	3052	8.959	0.000000
L occipital fusiform gyrus	-42	-65	-14	2537	8.133	0.000000
R inferior occipital gyrus	29	-86	-12	3249	9.711	0.000000
L inferior occipital gyrus	-28	-89	-3	4064	8.270	0.000000
R occipital (lingual gyrus)	27	-86	-3	4775	7.895	0.000000
L occipital (lingual gyrus)	-26	-89	-3	3451	7.565	0.000000
R middle occipital gyrus	35	-83	-9	7435	9.544	0.000000
L middle occipital gyrus	-40	-80	-9	8760	7.769	0.000000
L inferior frontal gyrus	-43	16	-6	10592	7.040	0.000002
R inferior frontal gyrus	47	37	12	387	6.004	0.000014
L middle frontal gyrus	-46	34	14	6029	6.671	0.000004
R middle frontal gyrus	50	28	24	3329	6.700	0.000004
L precentral frontal gyrus	-39	3	29	735	4.319	0.000465
R precentral frontal gyrus	38	4	26	885	3.839	0.001315
L frontal sub-gyral	-37	37	3	4058	5.900	0.000000
L inferior parietal lobe	-34	-56	39	194	2.600	0.018660
R superior parietal lobe	29	-59	43	439	3.758	0.001560
L superior parietal lobe	-34	-66	45	244	3.300	0.004220
L parietal (angular gyrus)	-34	-68	33	255	2.942	0.009110
R parietal (precuneus)	26	-62	36	1844	5.741	0.000020
L parietal (precuneus)	-22	-68	30	1542	4.395	0.000390
R temporal fusiform gyrus	32	-41	-18	4315	9.295	0.000000
L temporal fusiform gyrus	-43	-59	-15	4734	8.859	0.000000
R inferior temporal gyrus	46	-44	-18	269	3.897	0.001100
L inferior temporal gyrus	-51	-61	-10	1611	6.688	0.000000
R superior temporal gyrus	26	7	-36	283	4.227	0.000567
L superior temporal gyrus	-34	4	-12	3040	5.360	0.000052
R middle temporal gyrus	56	-41	-3	4687	4.610	0.000250
L middle temporal gyrus	-51	-59	-10	3275	5.728	0.000000
R insula	41	-5	0	1771	4.212	0.000500
L insula	-40	-2	-3	2407	5.749	0.000024
R amygdala	20	-8	-12	649	6.049	0.000010
L amygdala	-19	-5	-12	471	6.114	0.000010
R parahippocampal gyrus	23	-29	-18	4352	6.981	0.000000
L parahippocampal gyrus	-29	-28	-20	4414	9.904	0.000000
R uncus	28	-5	-19	501	3.850	0.001280
L hippocampus	-28	-14	-11	353	3.553	0.002400
L basal ganglia (putamen)	-22	-2	-6	3093	4.826	0.000158
R basal ganglia (lateral globus pallidus)	21	-1	-6	383	3.292	0.004305
L basal ganglia (lateral globus pallidus)	-19	-5	-6	923	5.105	0.000088
L basal ganglia (medial globus pallidus)	-15	-2	0	215	4.109	0.000700
R basal ganglia (putamen)	29.0	4.0	-6.0	1753	5.203	0.000072
R basal ganglia (caudate body)	11	4	12	279	2.675	0.016000
L basal ganglia (lateral globus pallidus)	-19.0	-5.0	-7.0	1315	5.379	0.000050
L thalamus	-1	-11	3	602	3.813	0.001390
L thalamus (medial dorsal nucleus)	-4	-14	6	443	3.896	0.001162

R thalamus (pulvinar)	8	-26	3	391	3.777	0.001503
L thalamus (pulvinar)	-10	-26	6	392	3.166	0.005642
L brainstem (midbrain)	-22	-29	-1	960	3.418	0.003270
R brainstem (midbrain)	14	-26	-3	1641	4.056	0.000820
L brainstem (pons)	-7	-26	-24	891	3.371	0.003600
R brainstem (pons)	11	-20	-30	1383	4.996	0.000100
L anterior cerebellum (culmen)	-26	-30	-21	6102	9.940	0.000000
R anterior cerebellum (culmen)	26	-41	-23	535	9.954	0.000000
L posterior cerebellum (declive)	-43	-65	-15	7764	8.647	0.000000
R posterior cerebellum (declive)	41	-59	-21	8209	7.497	0.000000
L posterior cerebellum (tuber)	-46	-62	-23	2087	7.110	0.000000
R posterior cerebellum (tuber)	44	-56	-23	1018	6.891	0.000000
L posterior cerebellum (uvula)	-28	-77	-23	1366	4.159	0.000600
R posterior cerebellum (uvula)	26	-68	-26	1055	4.044	0.000800
R posterior cerebellum (pyramis)	26	-68	-27	699	4.190	0.000600
R posterior cerebellum (cerebellar tonsil)	50	-50	-33	557	2.981	0.008300
R anterior cerebellum	26	-41	-23	535	7.011	0.000000
R claustrum	35	-5	0	467	3.899	0.001100
L claustrum	-34	-5	-6	547	4.467	0.000339
**Periphery > Centre**						
R occipital (cuneus)	11	-80	6	2863	-4.146	0.000670
L occipital (cuneus)	-1	-89	21	3060	-4.536	0.000292
R occipital (lingual gyrus)	11	-80	5	1238	-4.128	0.000700
L occipital (lingual gyrus)	-10	-86	0	723	-3.426	0.003220
R anterior cerebellum (culmen)	14	-60	-6	509	-3.230	0.004923

The table displays a selection of some of the relevant regions found. All contrasts were performed at p<.05 using cluster threshold correction. X, Y and Z represent Talairach coordinates. R, right; L, left. Only clusters respecting the minimum cluster size (190 voxels) were reported.
