# Supplementary material for: The Distinct Role of the Amygdala, Superior Colliculus and Pulvinar in Processing of Central and Peripheral Snakes
Source: PLoS One. 2015 Jun 15;10(6):e0129949. doi: 10.1371/journal.pone.0129949 (PMC4467980; doi:10.1371/journal.pone.0129949)
Supplement: S4 Table — (PDF) [file pone.0129949.s005.pdf]

S4 Table. Summary of random-effects (RFX)-GLM for Spatial location comparisons within Task: contrasts, outputs and statistics.

Region	Peak X (TAL)	Peak Y (TAL)	Peak Z (TAL)	Nr of voxels	*t*	*p*
**Implicit threat snake identification: Centre > Periphery**						
R occipital lobe (cuneus)	18	-93	-2	1003	5.094538	0.000060
L occipital lobe (cuneus)	-27	-92	-2	604	5.868786	0.000010
R occipital lobe (fusiform gyrus)	33	-82	-11	1695	7.058521	0.000000
L occipital lobe (fusiform gyrus)	-29	-49	-10	1473	3.062618	0.006400
R inferior occipital gyrus	32	-83	-11	1327	7.297149	6.3869e-07
L inferior occipital gyrus	-35	-80	-6	2116	7.073607	9.9115e-07
R middle occipital gyrus	28	-84	-7	1572	6.547256	0.000000
L middle occipital gyrus	-48	-64	-9	2629	6.428772	0.000000
R inferior temporal gyrus	63	-50	-7	336	4.190302	0.000490
L inferior temporal gyrus	-49	-56	-14	870	6.758980	0.000000
R occipital lobe (lingual gyrus)	19	-94	-7	1516	5.362890	0.000030
L occipital lobe (lingual gyrus)	-25	-72	-6	1315	2.665461	0.015280
R medial frontal gyrus	3	34	-11	373	2.984248	0.007620
L medial frontal gyrus	-6	47	0	459	2.199219	0.040400
L inferior frontal gyrus	-30	18	-19	572	3.800691	0.00120
R middle temporal gyrus	61	-50	-7	418	4.017759	0.000730
L middle temporal gyrus	-52	-56	-7	395	4.954580	0.000080
R temporal lobe (fusiform gyrus)	41	-53	-18	2428	6.754822	0.000000
L temporal lobe (fusiform gyrus)	-44	-56	-15	2560	8.634770	5.2947e-08
L limbic lobe (anterior cingulate)	-2	40	-6	1413	4.064604	0.000661
R limbic lobe (anterior cingulate)	2	37	-3	1597	4.251100	0.000432
R limbic lobe (parahippocampal gyrus)	26	-38	-14	285	5.277489	0.000000
L limbic lobe (parahippocampal gyrus)	-31	-31	-19	983	6.529417	0.000000
R amygdala	29	-2	-18	443	3.895133	0.000970
L amygdala	-22	-5	-11	317	4.460301	0.000260
L hippocampus	-31	-17	-11	214	3.405360	0.002900
R hippocampus	33	-17	-18	1635	3.604589	0.001800
R uncus	32	-18	-27	715	4.848818	0.000100
R basal ganglia (lateral globus pallidus)	17	-2	-6	186	3.189732	0.004824
L basal ganglia (lateral globus pallidus)	-19	-5	-6	563	4.097396	0.000613
L basal ganglia (medial globus pallidus)	-13	-2	0	272	4.236487	0.000400
L basal ganglia (caudate head)	-10	19	-3	787	4.003177	0.000761
R basal ganglia (caudate head)	11	19	-6	373	4.280343	0.000404
L basal ganglia (putamen)	-31	-17	-6	715	3.222891	0.004470
R basal ganglia (putamen)	28	0	-6	213	3.846591	0.001088
L anterior cerebellum (culmen)	-34	-38	-21	3521	6.917804	0.000000
R anterior cerebellum (culmen)	26	-38	-21	4567	6.702982	0.000000
L posterior cerebellum (uvula)	-31	-77	-23	700	3.754795	0.001300
L posterior cerebellum (tuber)	-46	-73	-23	872	3.963107	0.000800
R posterior cerebellum (declive)	29	-62	-12	5528	6.188478	0.000000

L posterior cerebellum (declive)	-47	-74	-18	6756	7.381061	5.4251e-07
**Implicit threat snake identification: Periphery > Centre**						
L occipital lobe (cuneus)	-4	-89	22	5378	-6.046895	0.000008
R occipital lobe (cuneus)	5	-89	22	5602	-6.166341	0.000006
R occipital lobe (precuneus)	14	-67	30	320	-3.969875	0.000821
L occipital lobe (lingual gyrus)	-10	-71	-3	1969	-4.359843	0.000337
R occipital lobe (lingual gyrus)	11	-69	-6	1861	-6.662436	0.000002
R superior occipital gyrus	38	-73	29	508	-3.006509	0.007256
L medial frontal gyrus	-7	-8	54	359	-3.242705	0.004283
R middle frontal gyrus	36	-10	45	368	-3.728714	0.001423
R middle frontal gyrus	29	34	34	3668	-5.993197	0.000009
R superior frontal gyrus	29	34	33	2350	-6.295620	0.000005
L frontal lobe (paracentral lobule)	-4	-23	47	192	-3.009200	0.007213
R limbic lobe (posterior cingulate)	11	-68	17	80	-2.883003	0.009528
R frontal lobe (precentral gyrus)	38	-20	51	1644	-4.565792	0.000211
L frontal lobe (precentral gyrus)	-31	-17	67	429	-2.797865	0.011477
R parietal lobe (precuneus)	0	-56	51	6281	-4.743000	0.000142
L parietal lobe (precuneus)	-4	-64	45	5060	-5.377595	0.000034
L superior parietal lobule	-11	-65	53	291	-3.870307	0.001030
R superior parietal lobule	8	-62	57	1027	-4.440924	0.000280
R parietal lobe (postcentral gyrus)	41	-23	48	1096	-4.586262	0.000202
R inferior parietal lobule	44	-65	43	1273	-2.405386	0.026506
R parietal lobe (supramarginal gyrus)	53	-49	30	473	-3.230610	0.004401
R superior temporal gyrus	63	-34	21	546	-2.205446	0.039940
R middle temporal gyrus	44	-74	29	437	-3.533256	0.002221
R temporal lobe (angular gyrus)	41	-74	33	334	-4.003207	0.000761
R posterior cerebellum (declive)	11	-70	-10	1014	-4.212409	0.000400
L posterior cerebellum (declive)	-7	-68	-11	1188	-3.528686	0.002200
L anterior cerebellum (culmen)	-7	-68	-6	1587	-4.395423	0.000311
R anterior cerebellum (culmen)	11	-68	-7	2185	-6.734857	0.000002
**Explicit threat detection: Centre > Periphery**						
L inferior frontal gyrus	-52	19	22	2788	4.602074	0.000254
R inferior frontal gyrus	44	4	27	2618	3.613911	0.002143
L middle frontal gyrus	-52	17	26	2200	3.455219	0.003024
R middle frontal gyrus	54	13	33	2513	2.839725	0.011317
L precentral gyrus	-43	5	34	295	2.584766	0.019278
R precentral gyrus	44	6	34	847	2.358749	0.030567
L occipital lobe (cuneus)	-22	-78	30	538	2.183130	0.043330
R occipital lobe (cuneus)	23	-78	33	1056	2.171469	0.044340
L occipital lobe (fusiform gyrus)	-28	-47	-10	1420	3.365031	0.003600
R occipital lobe (fusiform gyrus)	29	-49	-10	1606	4.120771	0.000700
L inferior occipital gyrus	-40	-80	-6	1951	5.091252	0.000090
R inferior occipital gyrus	36	-80	-3	1388	6.868035	0.000000

L occipital lobe (lingual gyrus)	-19	-98	-6	1518	4.864203	0.000140
R occipital lobe (lingual gyrus)	17	-42	-2	1985	2.319329	0.033080
R middle occipital gyrus	42	-65	9	1878	2.790018	0.012567
R inferior parietal lobule	33	-58	43	2087	2.790244	0.012560
R parietal lobe (postcentral gyrus)	57	-27	48	759	3.497622	0.002750
L parietal lobe (precuneus)	-22	-67	34	407	2.425387	0.026710
R parietal lobe (precuneus)	24	-71	38	1036	4.293588	0.000490
R superior parietal lobule	26	-73	43	312	3.426344	0.003210
L middle temporal gyrus	-45	-63	0	2268	2.148307	0.046393
L temporal lobe (fusiform gyrus)	-46	-61	-15	2860	8.381323	1.9252e-07
R temporal lobe (fusiform gyrus)	39	-47	-18	2522	5.699686	0.000000
R inferior temporal gyrus	45	-45	-18	290	4.205323	0.000500
L middle temporal gyrus	-49	8	-18	436	2.507057	0.022619
R middle temporal gyrus	41	-75	13	1181	3.356398	0.003740
L superior temporal gyrus	-46	13	-7	1058	4.340675	0.000444
R superior temporal gyrus	65	-26	16	668	3.567780	0.002360
L superior temporal gyrus (frontal lobe)	-46	12	-6	305	4.316106	0.000468
L insula	-29	19	-6	893	2.691607	0.015443
R insula	32	24	0	1469	3.956552	0.001000
L parahippocampal gyrus	-24	-32	-18	1668	5.721510	0.000000
R Parahippocampal gyrus	29	-33	-18	2054	5.085403	0.000000
L amygdala	-19	-5	-12	457	5.146481	0.000080
R amygdala	20	-8	-12	375	4.807897	0.000160
L basal ganglia (lateral globus pallidus)	-22	-5	-6	631	4.177834	0.000631
L basal ganglia (putamen)	-22	-2	-6	646	3.786168	0.001474
R basal ganglia (putamen)	30	-2	0	550	3.502614	0.002700
L thalamus (pulvinar)	-7	-26	3	352	3.354929	0.003757
R thalamus (pulvinar)	11	-29	3	442	4.086651	0.000769
L thalamus (medial dorsal nucleus)	-2	-14	3	365	3.195055	0.005304
R thalamus (medial dorsal nucleus)	2	-14	3	254	4.041580	0.000847
L thalamus	-1	-13	3	466	3.283177	0.004387
R thalamus	11	-32	3	504	3.916104	0.001112
L brainstem (pons)	-19	-28	-19	513	3.303902	0.004100
R brainstem (pons)	20	-30	-23	1654	3.773448	0.001500
R brainstem (medulla)	5	-20	-36	227	3.043032	0.007300
L brainstem (midbrain)	-15	-20	-12	2108	4.237364	0.000500
R brainstem (midbrain)	8	-26	2	1701	3.784672	0.001479
L anterior cerebellum	-21	-31	-23	210	3.499149	0.002700
R anterior cerebellum	23	-33	-23	818	4.443421	0.000300
L anterior cerebellum (culmen)	-22	-35	-18	5679	6.055143	0.000000
R anterior cerebellum (culmen)	26	-32	-21	7579	6.278596	0.000000
R posterior cerebellum (cerebellar tonsil)	20	-44	-31	583	3.227093	0.004900
L posterior cerebellum (declive)	-46	-65	-18	7563	7.786592	5.2639e-07
R posterior cerebellum (declive)	38	-77	-15	7938	7.364630	0.000000
L posterior cerebellum (pyramis)	-22	-74	-27	163	2.936316	0.009200
R posterior cerebellum (pyramis)	26	-68	-27	519	3.671604	0.001800

L posterior cerebellum (tuber)	-46	-62	-23	2019	5.860084	0.000000
R posterior cerebellum (tuber)	44	-56	-23	1557	4.652817	0.000200
L posterior cerebellum (uvula)	-37	-71	-23	1374	3.547452	0.002400
R posterior cerebellum (uvula)	26	-68	-26	1296	3.623112	0.002100
L claustrum	-34	-2	-6	362	3.182125	0.005453
R claustrum	35	-2	0	552	3.801648	0.001400

All contrasts were performed at p<.05 using cluster threshold correction. X, Y and Z represent Talairach coordinates. R, right; L, left. Only clusters respecting the minimum cluster size (implicit task: 170 voxels; explicit task: 153 voxels) were reported.
